# An Aggressively Enlarging Benign Mixed Epithelial Stromal Tumor (MEST) of the Kidney

**DOI:** 10.7759/cureus.31475

**Published:** 2022-11-14

**Authors:** W. C. Ian Janes, David Harvey

**Affiliations:** 1 Urology, Memorial University of Newfoundland, St. John's, CAN

**Keywords:** retroperitoneal tumor, hormonal imbalance, kidney, renal neoplasm, mest

## Abstract

Renal neoplasms represent a diverse spectrum of tumors with varying degrees of solid and cystic components that often dictate the potential for malignancy. Mixed epithelial stromal tumors (MEST) of the kidney represent an uncommon and heterogenous subset of these tumors, for which there remains a lack of extant literature and a poor degree of relative understanding. Our case represents a typical presentation of this uncommon neoplasm that contains several important differences regarding immunohistochemical staining and familial interplay that contribute to the literature surrounding these tumors.

## Introduction

Medical literature describes a variety of diverse renal tumors with variable patterns of aggressiveness and malignant potential [1]. Mixed epithelial stromal tumors (MEST) of the kidney are a rare subset of benign renal neoplasms first described approximately two decades ago [2]. Despite increasing recognition, there remain a few cases reported in the literature with approximately 100 extant reports [2-4]. These tumors, typically presenting in peri- and postmenopausal females, are characterized by their biphasic solid and cystic composition with variable growth patterns and cellularity [2]. Using current radiological techniques, the classification of malignancy for these entities is difficult and often warrants resection via partial or radical nephrectomy [5,6]. Here, we describe the case of a morphologically benign MEST of the kidney with an aggressive growth pattern and significant overall size.

## Case presentation

**Figure 1:**
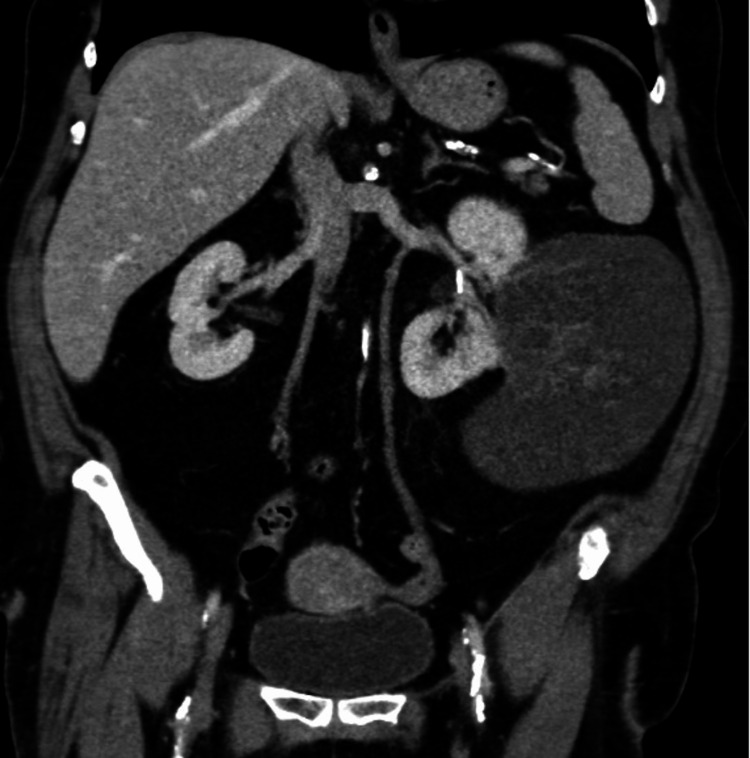
Unenhanced coronal CT scan demonstrating a large heterogenous left exophytic renal mass CT: computed tomography

A 59-year-old female with a history of surgically managed ovarian cysts presented with a several-month history of diffuse abdominal pain radiating to the left upper back and associated intermittent nausea. Further history elicited an absence of hematuria; however, an approximate 10-pound weight loss due to decreased oral intake was noted. The patient underwent imaging via computed tomography (CT) scanning with demonstrated findings of a large 16.5 cm (16.5 × 12.0 × 13.6 cm) well-circumscribed exophytic left renal mass. While predominantly cystic in composition, the mass contained multiple thickened internal septations and internal loculations along the medial and superior aspects. A small portion of the mass extended into the mid-renal sinus, while the lateral aspect abutted the abdominal wall along with a loop of the proximal descending colon without evidence of invasion. Faint internal enhancement and calcifications were seen, consistent with a Bosniak IV type cyst and of concern for cystic renal cell carcinoma (RCC) (Figure 1). These findings prompted an urgent referral to urology, where the patient subsequently opted for a left-sided retroperitoneal open radical nephrectomy using a modified subcostal incision to address the enlarging 16.5-cm mass. The procedure was uncomplicated, and the patient had an uneventful recovery.

Pathological examination revealed an approximate growth of 2 cm relative to the most recent CT scan, defining a well-encapsulated 18.5 cm (18.5 × 16.2 × 11.5 cm) mass with multiple cystic spaces of varying sizes divided by fibrous septa and lined by hobnail cells. The tumor contained yellow-brown serous fluid along with a yellow gelatinous material. Within the large mass was a smaller complex multiloculated cystic area 12.5 × 7.0 × 6.5 cm in size. The tumor demonstrated weak to strong nuclear expression for estrogen receptors (ER) and progesterone receptors (PR), respectively, indicative of an ovarian-type stroma with small groups of cells identified within. By immunohistochemical investigations, the stromal cells demonstrated the expression for inhibin, human melanoma black 45 (HMB45), melanoma-associated antigen recognized by T-cells (MART-1), and calretinin, supportive of steroidogenic origin. The tumor showed no features of malignancy, and the patient recovered without complication. Of interest to this case is the presentation of the patient’s sister with advanced RCC approximately six months following our MEST diagnosis.

## Discussion

MEST of the kidney are distinctive benign lesions accounting for less than 1% of all diagnosed renal neoplasms and require differentiation from other cystic tumors [3,7]. Our case represents a typical presentation of this uncommon neoplasm but contains several important differences that contribute to the literature surrounding these tumors. Preoperative diagnosis of MEST remains difficult to obtain radiologically given the delayed enhancement of the solid component of various complex cystic renal tumors during the nephrogenic phase of contrast-enhanced CT scanning [4]. Despite their generally benign nature, MEST are commonly treated via surgical intervention and pathologically diagnosed postoperatively [8]. Arguments for surveillance have been presented; however, the lack of radiological differentiation paired with clinical symptomatology and patient preference makes such undertakings difficult [6,9].

MEST are typically characterized as biphasic septated cystic growths containing diverse morphologies that range from predominantly cystic to mainly solid in nature [2,6]. The tumor reported in our case grossly measured 18.5 cm on pathological examination and represents a distinct entity as a few cases larger than this have been reported [10-12]. MEST tend to be noninvasive, well-circumscribed neoplasms, ranging in size from 2 cm to 24 cm, with an average of 6 cm and rarely extending beyond 10 cm [5,10,11]. The aggressive nature of our patient’s MEST, evidenced by the approximate 2-cm growth compared with the CT findings six weeks prior, represented an atypical presentation not previously documented.

On immunohistochemical profiling, the mesenchymal component of MEST is typically negative for melanocytic markers while displaying smooth muscle differentiation and immune reactivity for ER and PR [6,13]. Positivity for calretinin, cluster of differentiation 10 (CD10), and inhibin has also frequently been noted in prior reported cases and is indicative of steroidogenic origin [6,14]. A prominent theory postulated to explain the pathogenesis of these tumors centers around the altered hormonal milieu in peri- and postmenopausal females [4,10]. This hypothesis derives support from the distinctive ER and PR expression, indicating that tumorigenesis may be impacted by hormonal imbalances. Our case may provide further support for this explanation, given that the past medical history of ovarian cysts is shown to have derived from similar origins of hormonal hyperstimulation [15].

While melanocytic markers are typically negative when staining for MEST, our case displayed atypical HMB45 positivity, indicating the importance of considering findings in the context of radiological and morphological interpretations. Comparatively, one study found a lack of inhibin expression among 53 cases of MEST, while another report indicated 42% positivity for this marker among 14 cases [5,14]. In this examination of 14 cases, the expressions for ER and PR were reported as 62% and 85%, respectively [14]. These findings provide further evidence of the diversity of these tumors and support the necessity for holistic interpretation of these biphasic neoplasms. Understanding of these tumors is further complicated by recent reports of malignant transformation among MEST, believed to be related to the absence of immune reactivity to ER [7,12,13]. Our pathological specimen demonstrated weak reactivity for ER, while the patient had a sister with a diagnosis of RCC within the same calendar year, possibly indicating a greater potential for malignancy. While these two tumors may be independent occurrences, they propose a need for greater examination of a genetic component among individuals with MEST.

## Conclusions

MEST represent a rare benign tumor of the kidney with a strong female preponderance, likely resultant of hormonal hyperstimulation. These tumors require differentiation from other cystic renal neoplasms; however, this is difficult on current imaging techniques prompting urgent surgical management. While nephron-sparing surgery should be considered in feasible cases, these tumors are complex and still not widely understood. Our findings contribute to the growing literature surrounding MEST given the substantial size and aggressiveness of the reported tumor and the interesting possibility for familial interplay.
